# Mesenchymal stem cells reverse disease-specific abnormalities in nociceptive regions of the brain

**DOI:** 10.1093/braincomms/fcaf494

**Published:** 2025-12-15

**Authors:** Ryunosuke Fukushi, Masanori Sasaki, Hisashi Obara, Kota Kurihara, Ryosuke Hirota, Tomonori Morita, Atsushi Teramoto, Toshihiko Yamashita, Andrew M Tan, Stephen G Waxman, Jeffery D Kocsis, Osamu Honmou

**Affiliations:** Department of Orthopaedic Surgery, Sapporo Medical University School of Medicine, Sapporo, Hokkaido 060-8556, Japan; Department of Neural Regenerative Medicine, Institute of Regenerative Medicine, Sapporo Medical University School of Medicine, Sapporo, Hokkaido 060-8556, Japan; Department of Neural Regenerative Medicine, Institute of Regenerative Medicine, Sapporo Medical University School of Medicine, Sapporo, Hokkaido 060-8556, Japan; Division of Neuroscience, Department of Physiology, Sapporo Medical University School of Medicine, Sapporo, Hokkaido 060-8556, Japan; Department of Neurology, Yale University School of Medicine, New Haven, CT 06510, USA; Department of Orthopaedic Surgery, Sapporo Medical University School of Medicine, Sapporo, Hokkaido 060-8556, Japan; Department of Neural Regenerative Medicine, Institute of Regenerative Medicine, Sapporo Medical University School of Medicine, Sapporo, Hokkaido 060-8556, Japan; Department of Orthopaedic Surgery, Sapporo Medical University School of Medicine, Sapporo, Hokkaido 060-8556, Japan; Department of Neural Regenerative Medicine, Institute of Regenerative Medicine, Sapporo Medical University School of Medicine, Sapporo, Hokkaido 060-8556, Japan; Department of Orthopaedic Surgery, Sapporo Medical University School of Medicine, Sapporo, Hokkaido 060-8556, Japan; Department of Neural Regenerative Medicine, Institute of Regenerative Medicine, Sapporo Medical University School of Medicine, Sapporo, Hokkaido 060-8556, Japan; Department of Orthopaedic Surgery, Sapporo Medical University School of Medicine, Sapporo, Hokkaido 060-8556, Japan; Department of Neural Regenerative Medicine, Institute of Regenerative Medicine, Sapporo Medical University School of Medicine, Sapporo, Hokkaido 060-8556, Japan; Department of Orthopaedic Surgery, Sapporo Medical University School of Medicine, Sapporo, Hokkaido 060-8556, Japan; Department of Orthopaedic Surgery, Sapporo Medical University School of Medicine, Sapporo, Hokkaido 060-8556, Japan; Department of Neurology, Yale University School of Medicine, New Haven, CT 06510, USA; Neurorehabilitation Research Center, VA Connecticut Healthcare System, West Haven, CT 06516, USA; Department of Neurology, Yale University School of Medicine, New Haven, CT 06510, USA; Neurorehabilitation Research Center, VA Connecticut Healthcare System, West Haven, CT 06516, USA; Department of Neuroscience, Yale University School of Medicine, New Haven, CT 06510, USA; Department of Pharmacology, Yale University School of Medicine, New Haven, CT 06510, USA; Department of Neurology, Yale University School of Medicine, New Haven, CT 06510, USA; Neurorehabilitation Research Center, VA Connecticut Healthcare System, West Haven, CT 06516, USA; Department of Neuroscience, Yale University School of Medicine, New Haven, CT 06510, USA; Department of Neural Regenerative Medicine, Institute of Regenerative Medicine, Sapporo Medical University School of Medicine, Sapporo, Hokkaido 060-8556, Japan; Department of Neurology, Yale University School of Medicine, New Haven, CT 06510, USA

**Keywords:** bone marrow mesenchymal stem cells, spinal cord injury, dendritic spines, neuropathic pain, peripheral nerve injury

## Abstract

Neuropathic pain is characterized by hyperalgesia, allodynia or spontaneous pain arising from lesions or pathology in the somatosensory nervous system. Multiple mechanisms contribute to this pain following peripheral nerve and spinal cord injuries. Evidence shows that injury-induced changes in dendritic spine morphology in the dorsal horn may contribute to neuropathic pain presentation. Dendritic spines, critical postsynaptic structures for synaptic transmission, undergo remodelling from filopodia-like structures to mature, mushroom-shaped spines in nociceptive spinal cord regions after injury. Recent evidence indicates that peripheral nerve and spinal cord injuries affect local tissues and also lead to pathology in supraspinal brain regions. Interestingly, different injuries appear to target specific brain regions, potentially causing corresponding remodelling of dendritic spines. To investigate this, we examined whether spared nerve injury, as a peripheral nerve injury model, and spinal cord injury induce morphological changes in dendritic spines in different brain regions and whether systemic administration of mesenchymal stem cells could alleviate neuropathic pain by altering dendritic spine morphology. Our results demonstrate that both injuries induce significant morphological changes in dendritic spines in the brain and spinal cord. Specifically, the peripheral nerve injury model increases the density of mushroom-shaped spines in superficial Lamina II of the dorsal horn, whereas spinal cord injury induces similar changes in deeper Lamina V. In the brain, the peripheral nerve injury model showed increased mushroom-shaped spines in the sensory cortex and ventral posterior complex of the thalamus. In contrast, the spinal cord injury model showed these changes primarily in the thalamic intralaminar nuclei. Infused mesenchymal stem cells partially alleviated neuropathic pain in both models and reduced the density of mushroom-shaped spines in the respective affected regions. Gene expression analysis of cytoskeletal genes related to actin associated with the α-amino-3-hydroxy-5-methyl-4-isoxazolepropionic acid (AMPA) receptor (AKAP5, ACTR2, and SORBS2) revealed upregulation of these genes in the sensory cortex (in the peripheral nerve injury model) and the thalamus (in the spinal cord injury model). Mesenchymal stem cells suppressed these upregulations, which were associated with reduced neuropathic pain. These findings suggest that infused mesenchymal stem cells can protect against the abnormal remodelling of dendritic spines, thereby contributing to pain alleviation regardless of injury type or affected region. The systemic administration of mesenchymal stem cells thus offers a promising therapeutic approach for treating multiple neuropathic pain conditions through structural and molecular alterations in dendritic spines.

## Teaser

Fukushi *et al*. study that intravenous infusion of mesenchymal stem cells reduces neuropathic pain by reversing abnormal dendritic spine structures in the spinal cord and, importantly, in the brain. This suggests a new way to treat neuropathic pain from peripheral nerve injury and spinal cord injury.

## Graphical Abstract

**Graphical Abstract:**
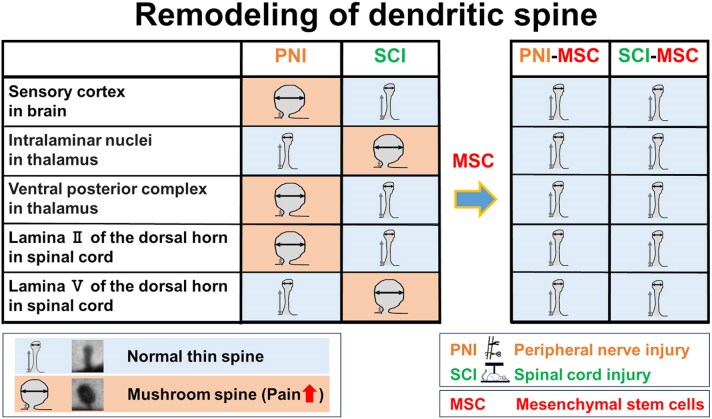

## Introduction

Neuropathic pain is caused by pathological changes in the somatosensory nervous system, often from injury manifesting as hyperalgesia, allodynia or spontaneous pain.^1^ However, the processes and mechanisms underlying neuropathic pain in patients with spinal cord or peripheral nerve injury (PNI) are complex and are not fully understood. Dendritic spines are micron-sized structures protruding from the dendrites of neurones and receive synaptic inputs from other neurones. Dendritic spines are found throughout the central nervous system (CNS) and are very plastic in that their size and shape can change markedly in response to neural activity.^2^ Emerging evidence has demonstrated a strong structure–function relationship between abnormal alterations of dendritic spines in the spinal cord dorsal horn and the presentation of neuropathic pain following peripheral nerve injury^3,4^ and spinal cord injury (SCI).^5,6^ Especially, injury and disease-associated dendritic spine changes have been referred to as ‘dendritic spine dysgenesis’, which includes an increase in a mushroom-shaped dendritic spine density along with other common structural motifs associated with nociceptive hyperexcitability.^4,6^ Recent studies have shown that spinal cord and peripheral nerve injuries cause extensive damage to distant, functionally connected brain cells as well as locally injured cells.^7^ However, few studies have examined the morphology of dendritic spines in these brain regions after either PNI or SCI to determine if spine changes occur in supraspinal brain areas related to pain processing.

Notably, after PNI or SCI, pain processing pathways diverge to affect different brain regions associated with nociception. Animal studies have demonstrated that pain following PNI is associated with increased neuronal activity in the ventral posterior thalamus^8^ and the sensory cortex (SI).^9,10^ In contrast, after SCI, increased neuronal activity associated with pain has been reported in the intralaminar nuclei of the thalamus,^11-14^ but not in the SI. A limitation of these studies is that rodent models cannot fully replicate the clinical features of human neuropathic pain.^15,16^ There have been no studies that have examined changes in dendritic spine morphology in the thalamus and SI following PNI and SCI in rat models.^17^

Intravenous infusion of mesenchymal stem cells (MSCs) has been shown to contribute to functional recovery after PNI^18-20^ and SCI.^21-25^ Analgesic effects of MSCs in neuropathic pain have been suggested in the literature.^26,27^ However, most delivery routes in the previous studies involved local or intrathecal injection, not intravenous, as used in the current study. The analgesic efficacy of intravenously administered bone marrow–derived MSC into PNI and SCI has not been well studied. Given these findings, we hypothesized that intravenous infusion of MSCs might mitigate neuropathic pain after both PNI and SCI. It has been reported that infused MSCs orchestrate a process that includes neuroprotection, stabilization of the disrupted blood–spinal cord barrier, angiogenesis, remyelination, axonal regeneration, remote effects, induction of neural plasticity,^28^ neuroimmune^29^ and secretome^30^ modulation.

The interaction between neuropathic pain and changes in dendritic spine morphology in the SI and thalamus following intravenous MSC infusion has not been explored. Such an interaction would be especially relevant given the mounting body of evidence showing that correcting abnormal dendritic spine plasticity can reduce injury-induced pathological pain.^4,31^ In this study, we hypothesized that MSC infusion at Day 3 might also reverse pathological dendritic spine morphology, i.e. ‘dendritic spine dysgenesis’, thereby affecting the prevention of neuropathic pain establishment.

To this end, we investigated whether intravenous infusion of MSCs alleviates neuropathic pain and alters the morphology of dendritic spines in pain-related brain regions after PNI or SCI using two different animal models of neuropathic pain. For neuropathic pain induced by PNI, we used the spared nerve injury model in this study. For SCI-induced neuropathic pain, we employed the contusive SCI model in rats. We analysed the changes in anatomical structure and molecular signals associated with dendritic spine remodelling to understand pain-related pathways in the brain and spinal cord, both before and after the intravenous infusion of MSCs.

## Materials and methods

### Animals

All experiments were conducted in accordance with the institutional guidelines of Sapporo Medical University. The Animal Care and Use Committee of Sapporo Medical University approved the use of animals in this study.

### Preparation of rat MSC from rat bone marrow

MSCs were prepared and cultured according to our previous studies.^21,22,32^ Briefly, bone marrow obtained from the femoral bones of 10 adult (6–8 weeks old) Sprague–Dawley (SD) rats were diluted to 15 mL with Dulbecco’s modified Eagle’s medium (DMEM) (Sigma, St. Louis, MO, USA) supplemented with 10% heat-inactivated foetal bovine serum (FBS) (Thermo Fisher Scientific Inc., Waltham, MA, USA), 2 mM l-glutamine (Sigma), 100 U/mL penicillin, and 0.1 mg/mL streptomycin (Thermo Fisher Scientific Inc.) and incubated for 5–7 days at 37°C in a humidified atmosphere containing 5% CO_2_. When cultures almost reached confluence, the adherent cells were detached with 0.25% trypsin-ethylenediaminetetraacetic acid (EDTA) solution (Sigma) and subcultured at 1 × 10^4^ cells/mL of the medium. Just after two passages, MSCs were cryopreserved in CELLBAKER 1 (NIPPON ZENYAKU KOGYO CO., LTD., Fukushima, Japan). Cryopreserved cells were stored for 2–4 weeks until use. A previous phenotypic analysis of the surface antigens revealed clusters of differentiation (CD) 45^−^ CD73^+^ CD90^+^ and CD106^−^ on MSCs.^33^ MSCs were introduced via intravenous infusion 3 days after injury in both the PNI and SCI models.

### PNI model

Twenty adult male SD rats aged 8 weeks (250–300 g) were used for the PNI model. The rats were anaesthetized using an intraperitoneal injection of ketamine (90 mg/kg) and xylazine (4 mg/kg). The model was induced using the method described below.^34^ The left sciatic nerve was exposed at the midthigh level, and the tibial, common peroneal and sural nerves were identified. The tibial and peroneal nerves were ligated with 5-0 silk and cut. The sural nerve was spared from any lesion. The muscular layer and incision of the shaved skin layer were closed in layers using sutures. Rats were housed in an atmosphere of 50% humidity at a temperature of 24 ± 2°C. Intravenous infusion of MSC (*n* = 10) or vehicle (*n* = 10) was performed via right femoral veins.^21,22^ In PNI-sham animals (*n* = 10), we exposed only the sciatic nerve and its branches without nerve injury.

### SCI model

Twenty adult male SD rats aged 8 weeks (250–300 g) were used for the SCI model. Contusive SCI was performed as described previously.^5,21-23^ The rats were anaesthetized using an intraperitoneal injection of ketamine (90 mg/kg) and xylazine (4 mg/kg). Following the incision, the T9 vertebra was stabilized by clamping the rostral T8 and caudal T10 vertebral bodies with forceps, and dorsal laminectomy was performed at the T9–10 level. A 150 kDyn contusion was then delivered using an Infinite Horizon Impactor (IHI; Precision Systems and Instrumentation, LLC, Lexington, KY, USA). To ensure the invariant nature of our SCI procedure, we compared the actual injury force—following contusive impact—against the IHI protocol impact force. Here, there was a *<0.05% variation between these values, which confirmed the reliability of this method in agreement with others.^23,35^ Animals received daily postoperative care, including manual bladder expression twice daily, ensuring their welfare over the experimental period. Rats were housed in an atmosphere of 50% humidity at a temperature of 24 ± 2°C. Intravenous infusion of MSC (*n* = 10) or vehicle (*n* = 10) was performed via right femoral veins.^21,22^ SCI-sham animals (*n* = 10) underwent laminectomy only without contusive injury.

### Experimental protocols

**Figure 1:**
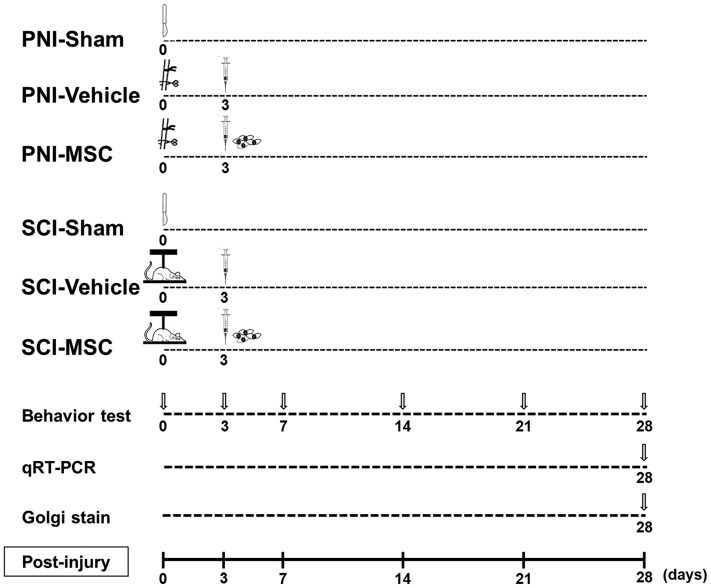
**Experimental protocol.** PNI-sham, PNI-vehicle, PNI-MSC, SCI-sham, SCI-vehicle and SCI-MSC groups were employed. MSCs were introduced via intravenous infusion 3 days after injury in both the PNI and SCI models. Behavioral evaluations were conducted on Days 0, 3, 7, 14, 21 and 28. Tissue samples were collected 28 days post-injury for Golgi staining and qRT-PCR. MSC, mesenchymal stem cell; PNI, peripheral nerve injury; qRT-PCR, quantitative reverse transcription-polymerase chain reaction; SCI, spinal cord injury.

The experimental protocol is illustrated in Fig. 1. In both PNI and SCI models, the animals were randomized and received a single intravenous infusion of MSCs at 1.0 × 10^6^ cells in 1.0 mL of fresh DMEM or vehicle (1.0 mL fresh DMEM alone) 3 days after model induction. Behavioral studies were performed at 0, 3, 7, 14, 21 and 28 days post-model inductions. Quantitative reverse transcription-polymerase chain reaction (qRT-PCR) and Golgi staining were performed at 28 days post-model inductions. All rats were injected daily with cyclosporine A (10 mg/kg, IP) for 1 day before intravenous infusion of MSCs or vehicle.^21,24^

### Locomotor function

Locomotor function was assessed in SCI animals using the Basso, Beattie and Bresnahan (BBB) open-field locomotor rating scale.^36^ The BBB score consists of combinations of hindlimb movements, trunk position and stability, stepping, coordination, paw placement, toe clearance and tail position. Rats were scored 2 days before SCI induction and 0, 3, 7, 14, 21 and 28 days post-SCI induction. In this study, all SCI animals displayed spinal shock, scoring 0 on the BBB rating scale on Day 1 after injury, and progressively improved function, reaching a BBB score of over 9 by Day 28.

### Evaluation of mechanical allodynia (von Frey test)

The rats were placed in a Plexiglas box (18 × 25 × 18 cm) on a wire mesh platform and allowed to acclimatize for 20 min before measurement to suppress excitation (*n* = 10/PNI-sham group, *n* = 10/PNI-vehicle group, *n* = 10/PNI-MSC group, *n* = 10/SCI-sham group, *n* = 10/SCI-vehicle group, *n* = 10/SCI-MSC group). Mechanical sensory thresholds were determined by paw withdrawal away from a series of graded von Frey filaments (Semmes I Weinstein Monofilaments, North Coast Medical Inc., San Jose, CA) applied to the glabrous surface of the paw. A modification of the Dixon ‘up–down’ method was used to determine the force value when paw withdrawal occurred 50% of the time, which was interpreted as the mechanical nociceptive threshold.^3^ In the PNI model, the mechanical nociceptive threshold (stimulus intensity) of the ipsi-lesional response (left; constricted) had a 50% likelihood of a paw withdrawal response (50% threshold). Thresholds on the ligated side (left side) were assessed. The mean stimulus intensities of five measurements of thresholds on the ligated side (left side) were evaluated.^3,37^ This assessment was conducted on Days 0, 3, 7, 14, 21 and 28. In the SCI model, the mechanical nociceptive threshold (stimulus intensity) of the averaged bilateral response had a 50% likelihood of a paw withdrawal response (50% threshold). Thresholds on the averaged bilateral response were assessed. This assessment was conducted on Day 28 in the SCI model.

### Evaluation of thermal hyperalgesia (radiant heat test)

The rats were placed in a Plexiglas box on a glass plate for 20 min before measurement to suppress excitation (*n* = 10/PNI-sham group, *n* = 10/PNI-vehicle group, *n* = 10/PNI-MSC group, *n* = 10/SCI-sham group, *n* = 10/SCI-vehicle group, *n* = 10/SCI-MSC group). Thermal hyperalgesia was assessed by measuring the latency of hind paw withdrawal in response to a radiant heat source. Briefly, the hind paw plantar area was stimulated using a heat source (Tail Flick Analgesia Meter, IITC Life Science Inc., Woodland Hills, CA), and the escape time was measured. The heat source was of sufficient intensity to allow the control rats to escape within 6–8 s, and a cut-off time of 10 s was used to avoid tissue damage. In the PNI model, the rats were stimulated five times each on the left and right sides, with at least 5-min intervals between the two stimuli. This assessment was conducted on Days 0, 3, 7, 14, 21 and 28. The mean withdrawal latencies of the five measurements were evaluated.^3,38^ In the SCI model, the rats were stimulated five times on both left and right sides, with at least 5-min intervals between the two stimuli. The mean withdrawal latencies of the five measurements were evaluated. This assessment was conducted on Day 28.

### Golgi staining

For Golgi-Cox staining with the use of a commercial kit and according to the manufacturer’s instructions (FD NeuroTechnologies, PK401, Ellicott, MD, USA), the rats (*n* = 4/PNI-sham group, *n* = 4/PNI-vehicle group, *n* = 4/PNI-MSC group, *n* = 4/SCI-sham group, *n* = 4/SCI-vehicle group, *n* = 4/SCI-MSC group) were killed using deep anaesthesia with ketamine (75 mg/kg) and xylazine (10 mg/kg, IP). Brain and spinal cord tissues were quickly removed and trimmed (under 5 min), rinsed in distilled water and immersed in the kit’s impregnation solution. After the incubation period (3 weeks), 200-μm-thick coronal sections were cut on a vibratome (DTK-1000 microslicer, Dosaka EM, Kyoto, Japan) and mounted on gelatinized glass slides. Sections were stained, rinsed in distilled water, dehydrated, cleared and glass cover slipped with a Permount mounting medium (#SP15-100-1, FALMA Co., Ltd., Tokyo, Japan).^3,5,39^

### Dendritic spine/image analysis

**Figure 2:**
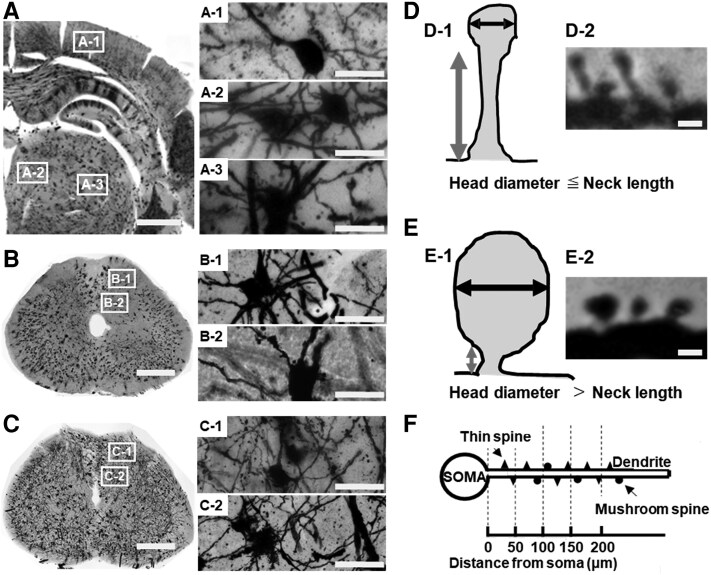
**Golgi-stained dendritic spines were examined in the coronal sections of the brain and spinal cord tissue.** (**A**) Golgi-stained coronal section of the brain. Measurements were performed in the SI (A-1), intralaminar nuclei of the thalamus (A-2) and a ventral posterior complex of the thalamus (A-3). (**B**) Golgi-stained coronal section of the spinal cord. In the PNI model, morphological changes were measured in Laminae II (B-1) and V (B-2) at the L5 segmental level of the spinal cord. In the SCI model, dendritic spine changes were measured in Laminae II (C-1) and V (C-2) at the Th7 segmental level of the spinal cord. (**D**) Thin-shaped spine, (D-1) illustration, (D-2) microscopic image, (**E**) mushroom-shaped spine, (E-1) illustration, (E-2) microscopic image, (**F**) Sholl analysis. Scale bars = 1.5 mm (**A**), 1 mm (**B** and **C**), 30 μm (A-1–A3, B-1 and B-2, C-1 and C-2), 1 μm (D-2 and E-2). Each data point represents the mean dendritic spine density per animal. PNI, peripheral nerve injury; SCI, spinal cord injury.

**Figure 3:**
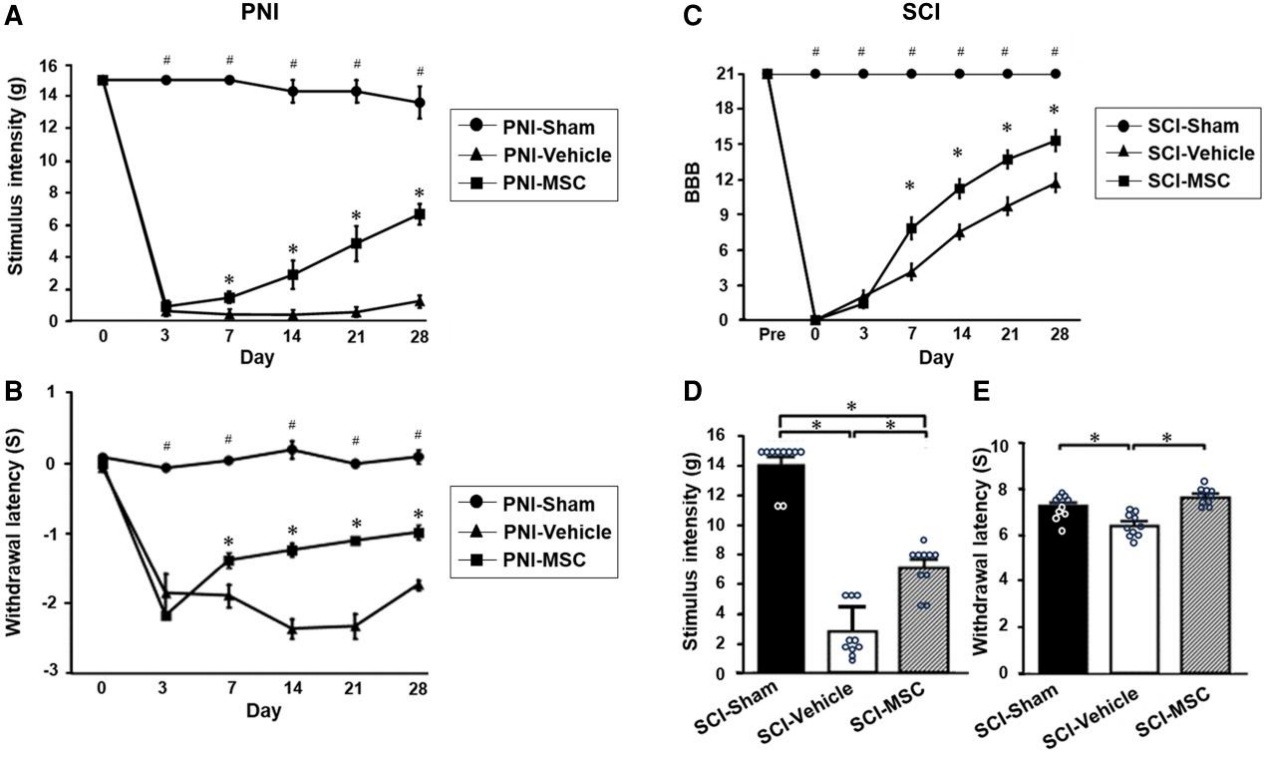
**Infused MSCs alleviate neuropathic pain induced by both PNI and SCI.** (**A**) The von Frey filament test of the PNI model, (**B**) the radiant heat test of the PNI model, (**C**) BBB score of the SCI model, ^#^*P* < 0.05 vehicle versus sham, *P < 0.05 vehicle versus MSC, (**D**) the von Frey filament test of the SCI model, (**E**) the radiant heat test of the SCI model. *P < 0.05, Data are presented as mean ± SEM. *N* = 10. Each data point represents the mean mechanical nociceptive threshold per animal for **A**, **B**, **D** and **E** and the mean BBB open-field locomotor rating scale score per animal for **C**. MSC, mesenchymal stem cell; PNI, peripheral nerve injury; SCI, spinal cord injury; SEM, standard error of the mean. One-way ANOVA, and the Tukey *post hoc* test was used for subgroup comparisons. Given the small size, we confirmed all results with the Kruskal–Wallis two-way analysis of variance followed by the Bonferroni *post hoc* test.

Golgi-stained slides of the brain (Fig. 2A) and spinal cords (Fig. 2B, L5 level for the PNI model; Fig. 2C, Th7 level for the SCI model) were photographed with a light microscope (BZ-X700, Keyence, Osaka, Japan). We sampled one neurone each from Layer V of the SI (Fig. 2A-1), intralaminar nuclei of the thalamus (Fig. 2A-2) and ventral posterior complex of the thalamus (Fig. 2A-3). Localization of hindlimb sensation in SI areas was at the following coordinates (Fig. 2A-1: 3.0 mm lateral; 3.0 mm posterior to the bregma) based on a previous study.^40^ In the spinal cord, we sampled one neurone each from Lamina II (Fig. 2B-1 for the PNI model, Fig. 2C-1 for the SCI model) and Lamina V (Fig. 2B-2 for the PNI model, Fig. 2C-2 for the SCI model) per animal (*n* = 4/group), respectively.

For extensive morphometric analysis, these Golgi-stained slides were examined by a light microscope (BX51, Olympus, Tokyo, Japan) equipped with an automatic stage and coupled to a computer with Neurolucida software (MicroBrightField, Colchester, VT, USA). Four morphological criteria of the sampled neurone as follows: (i) from one rat, neurones must be located within the SI, intralaminar nuclei, and ventral posterior complex of the thalamus, Laminae Ⅱ and Ⅴ of the dorsal horn, respectively; (ii) Golgi-stained neurones must have had dendrites and spines that were completely impregnated and appeared as a continuous length; (iii) dendritic arbours remained within the thickness of the tissue section; and (iv) the cell body diameter (dorsal–ventral) fell between 15 and 45 μm in the SI,^41^ thalamus,^42^ and spinal cord.^3,4^ Three-dimensional (3D) traces of the identified neurones were generated in Neurolucida software (MicroBrightField). Morphological measurements were performed with Neuroexplorer software (MicroBrightField). The criteria for identifying the dendritic spines were based on previous studies.^4-6,43,44^ The dendritic spines were classified into two types: thin and mushroom-shaped spines; i.e. thin spines were considered spines that had head-like enlargements with diameters less than the length of the neck (Fig. 2D; D-1, illustration; D-2, microscopic image), and mushroom spines were spines with head diameters greater than the length of the neck (Fig. 2E; E-1, illustration; E-2, microscopic image). Dendritic spine density was expressed as spine number per micrometre of dendrite length. To determine changes in spine distribution relative to the cell body among groups, a Sholl analysis was performed using the Neurolucida software (MicroBrightField). Spherical bins of 50 μm wide were formed around the cell body (soma), spine density was measured within each bin, data in each bin were averaged within each group, and the means were compared to equivalent bins across groups (Fig. 2F).

### Quantitative reverse transcription-polymerase chain reaction

Rats were euthanized using deep anaesthesia with ketamine (75 mg/kg) and xylazine (10 mg/kg, IP) (*n* = 6/PNI-sham group, *n* = 6/PNI-vehicle group, *n* = 6/PNI-MSC group, *n* = 6/SCI-sham group, *n* = 6/SCI-vehicle group, *n* = 6/SCI-MSC group). The SI and thalamus were dissected, frozen in liquid nitrogen and stored at −80°C until use. After homogenization, total RNA was purified using the RNeasy Plus mini kit (QIAGEN, Venlo, the Netherlands). RNA quality was assessed using the Bioanalyzer RNA 6000 nano kit (Agilent Technologies, Santa Clara, CA). Samples showing RNA integrity number (RIN) > 7.4 were used in this study. The SuperScript VILOTM cDNA Synthesis Kit (Invitrogen, Carlsbad, CA, USA) was used for qRT-PCR using 100 ng of mRNA input. TaqMan Universal Master Mix II with uracil-*N* glycosylase and TaqMan Gene Expression Assays for A-Kinase Anchor Protein 5 (*AKAP5; Rn01786021_m1)*, actin-related protein 2 (*ACTR2;* Rn01434079_m1), Arg-binding protein 2 (*SORBS2*; Rn00587190_m1) and glyceraldehyde 3-phosphate dehydrogenase (*GAPDH*; Rn01775763_g1) were purchased (Thermo Fisher Scientific Inc., Waltham, MA). qRT-PCR analysis was performed in triplicate using PRISM7500 with 7500 software v2.3 (Thermo Fisher Scientific Inc.). Thermal cycling was performed at 50°C for 2 min and 95°C for 10 min, followed by 40 cycles at 95°C for 15 s and 60°C for 1 min. The ΔCT was calculated against the endogenous control (GAPDH), and the ΔΔCT was calculated against the ΔCT of the sham. Fold change (FC) was calculated using the ΔΔCT method.^45^

### Statistical analysis

All statistical analyses were performed using Statistical Product and Service Solutions version 20 for Macintosh (SPSS Inc., Chicago, IL, USA). The Shapiro–Wilk test confirmed that all data were normally distributed. Accordingly, data were analysed using one-way ANOVA, and the Tukey *post hoc* test was used for subgroup comparisons. Given the small size, we confirmed all results with the Kruskal–Wallis two-way analysis of variance followed by the Bonferroni *post hoc* test. Data were expressed as the mean ± standard error of the mean (SEM). Differences were considered statistically significant at *P* < 0.05. Effect sizes for ANOVA were reported as *η*² (Eta squared) for the main effect and *d* (Cohen’s *d*) for *post hoc* tests, calculated using the general linear model procedure in SPSS.

## Results

### Infused MSCs alleviate neuropathic pain induced by PNI and SCI

PNI model: the paw withdrawal threshold (Fig. 3A) and thermal paw withdrawal latencies (Fig. 3B) decreased after PNI induction, and these did not improve in the PNI-vehicle group during the study period. However, the PNI-MSC group showed gradual increases in the paw withdrawal threshold (Fig. 3A) and thermal paw withdrawal latencies (Fig. 3B) with statistical significance on Days 7, 14, 21 and 28. (We infused MSCs or vehicle on Day 3 after PNI induction.) These results show that infused MSCs alleviate neuropathic pain induced by PNI.

SCI model: we used the BBB scale to measure locomotor function in the SCI model. Scores on the BBB in the SCI-MSC group were significantly higher than those of the SCI-vehicle group at 7, 14, 21 and 28 days after SCI induction, respectively (Fig. 3C). These results indicated that the infused MSCs provided motor functional recovery in a rat model of acute SCI, which is consistent with our previous studies.^22,23,25^

The paw withdrawal threshold (Fig. 3D) and thermal paw withdrawal latencies (Fig. 3E) decreased in the SCI-vehicle group on Day 28. However, the SCI-MSC group showed greater increases in the paw withdrawal threshold (Fig. 3D) and thermal paw withdrawal latencies (Fig. 3E) than the SCI-vehicle group with statistical significance on Day 28. These results indicate that infused MSCs alleviate neuropathic pain induced by SCI.

The von Frey test for mechanical allodynia and the radiant heat test for thermal hyperalgesia used in this study required the rats to have both paws touching the ground (BBB score > 9). Because SCI animals displayed a BBB of 9 or higher only on Day 28, we measured the paw withdrawal threshold (Fig. 3D) and thermal paw withdrawal latencies on Day 28.

### Dendritic spine morphology changes following PNI and SCI

**Figure 4:**
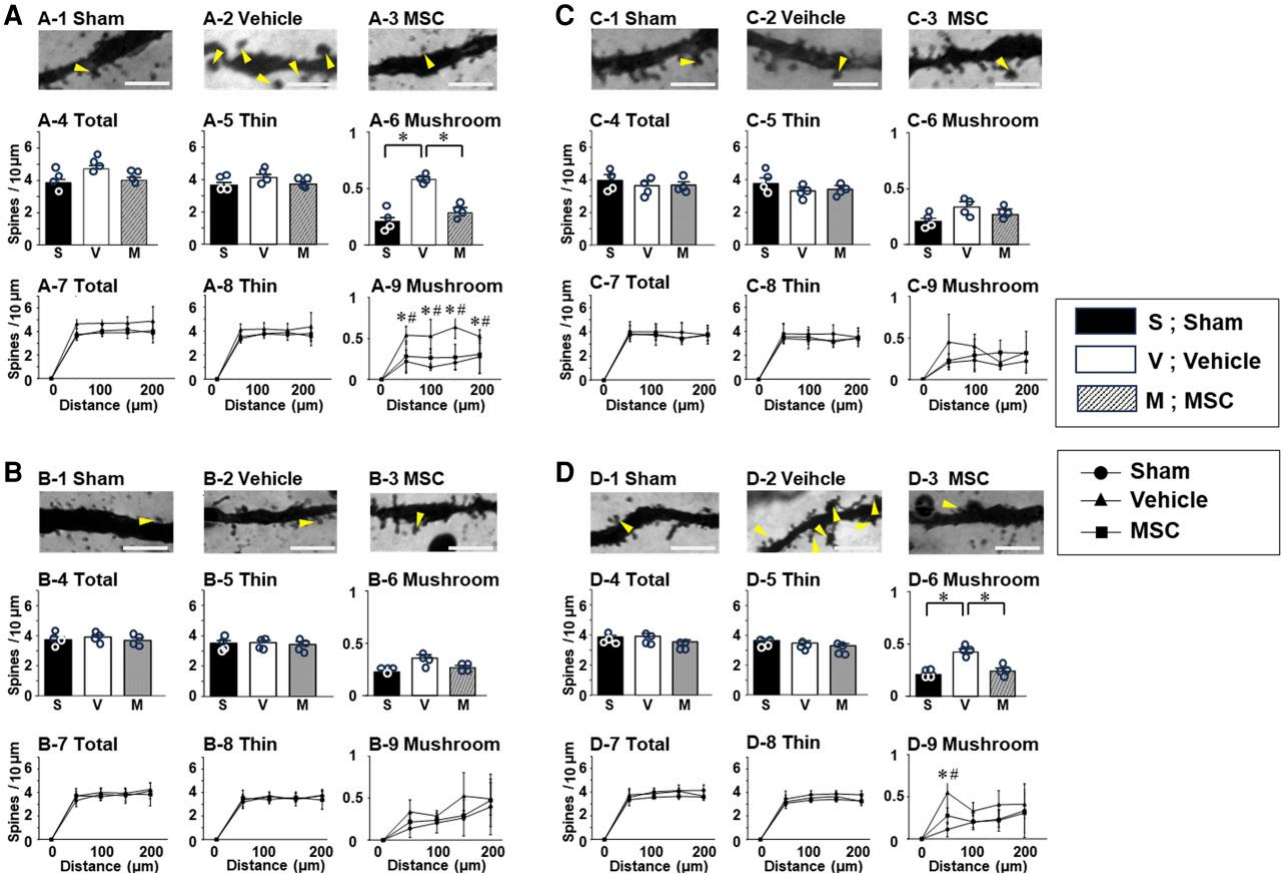
**Injury-specific remodelling of dendritic spines in Lamina II of the PNI model and Lamina V of the SCI model.** (**A**) PNI model, Lamina II of the dorsal horn; (**B**) SCI model, Lamina II of the dorsal horn; (**C**) PNI model, Lamina V of the dorsal horn; (**D**) SCI model, Lamina V of the dorsal horn. Representative Golgi-stained tissue of dendritic spines from sham (−1), vehicle (−2) and MSC (−3). Dendritic spine density is from total (−4), only thin- (−5) or mushroom-shaped spines (−6). Spatial distribution of dendritic spines using a Sholl analysis from total (−7), only thin- (−8) or mushroom-shaped spines (−9). Data are presented as mean ± SEM. *N* = 4. MSC, mesenchymal stem cells, *P < 0.05.(−6), ^#^*P* < 0.05 vehicle versus sham (−9), *P < 0.05 vehicle versus MSC (−9). Scale bars = 3 μm. Arrowheads indicate mushroom-shaped dendritic spines (dendritic spine dysgenesis). MSC, mesenchymal stem cell; PNI, peripheral nerve injury; SCI, spinal cord injury; SEM, standard error of the mean. One-way ANOVA, and the Tukey *post hoc* test was used for subgroup comparisons. Given the small size, we confirmed all results with the Kruskal–Wallis two-way analysis of variance followed by the Bonferroni *post hoc* test.

**Figure 5:**
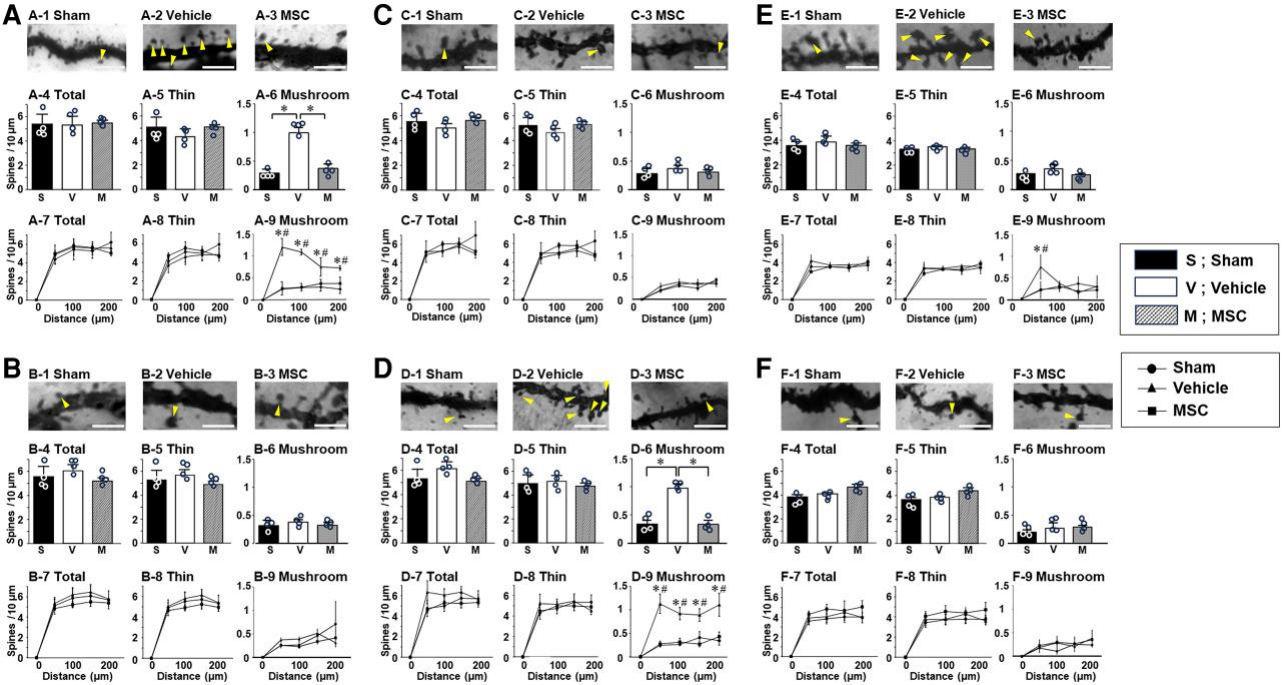
**Remodelling of dendritic spines in the SI and thalamic ventral posterior complex in the PNI model versus thalamic intralaminar nuclei of the SCI model.** (**A**) PNI model, SI; (**B**) SCI model, SI; (**C**) PNI model, intralaminar nuclei of the thalamus; (**D**) SCI model, intralaminar nuclei of the thalamus; (**E**) PNI model, ventral posterior complex of the thalamus; (**F**) SCI model, ventral posterior complex of the thalamus. Representative Golgi-stained tissue of dendritic spines from sham (−1), vehicle (−2) and MSC (−3). Dendritic spine density is from total (−4), only thin- (−5) or mushroom-shaped spines (−6). Spatial distribution of dendritic spines using a Sholl analysis from total (−7), thin- (−8) or only mushroom-shaped spines (−9), respectively. Data are presented as mean ± SEM. *N* = 4. MSC, mesenchymal stem cells, *P < 0.05 (−6), ^#^*P* < 0.05 vehicle versus sham (−9), *P < 0.05 vehicle versus MSC (−9). Scale bars = 3 μm. rrowheads indicate mushroom-shaped dendritic spines (dendritic spine dysgenesis). Each data point represents the mean dendritic spine density per animal. MSC, mesenchymal stem cell; PNI, peripheral nerve injury; SCI, spinal cord injury; SEM, standard error of the mean. One-way ANOVA, and the Tukey *post hoc* test was used for subgroup comparisons. Given the small size, we confirmed all results with the Kruskal–Wallis two-way analysis of variance followed by the Bonferroni *post hoc* test.

An increase in mushroom-shaped dendritic spine density in the spinal cord dorsal horn, i.e. Laminae II–V is a common morphological motif that appears following PNI^4^ and SCI^5^—and has been offered as a powerful morphological readout for neuropathic pain.^4,6^ In this study, we profiled dendritic spines in neurones in the spinal cord as well as the brain (SI and thalamus). The general morphology, including the number of spines, cell body dimensions (medial–lateral; dorsal–ventral) and number of primary dendrites at sampling, showed no difference across the groups (Supplementary Table 1). As shown in Fig. 4 (spinal cord) and Fig. 5 (brain), representative microscopic images of Golgi staining reveal spiny protrusions on dendrites with morphologies that qualitatively differ across the three experimental groups in this study. All *P*-values and effect sizes in Figs. 4 and 5 are provided in Supplementary Table 2.

**Figure 6:**
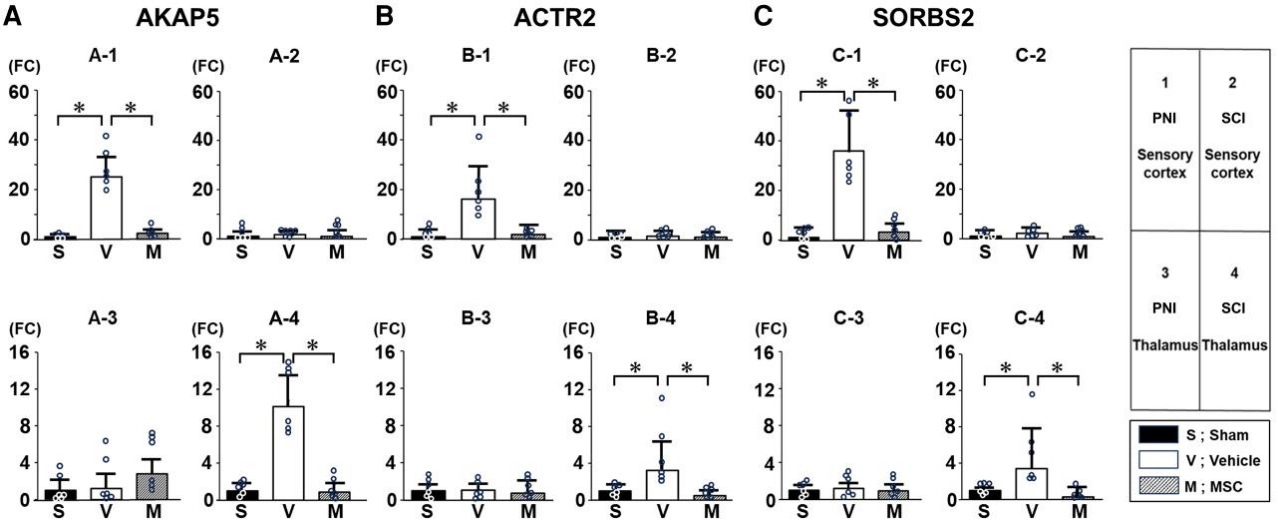
**Infused MSCs suppress an increase in AMPA-associated gene expressions selectively in the SI of the PNI model or the thalamus of the SCI model.** (**A**) AKAP5, (**B**) ACTR2, (**C**) SORBS2. mRNA expression of the SI of the PNI model (−1), the SI of the SCI model (−2), the thalamus of the PNI model (−3) and the thalamus of the SCI model (−4), respectively. Data are presented as mean ± SEM. *P < 0.05. *N* = 6. Each data point represents the mean FC per animal. *ACTR2*, actin-related protein 2; *AKAP5*, A-kinase anchor protein 5; AMPA, α-amino-3-hydroxy-5-methyl-4-isoxazolepropionic acid; MSC, mesenchymal stem cells; PNI, peripheral nerve injury; SCI, spinal cord injury; SEM, standard error of the mean; *SORBS2*, Arg-binding protein 2. One-way ANOVA, and the Tukey *post hoc* test was used for subgroup comparisons. Given the small size, we confirmed all results with the Kruskal–Wallis two-way analysis of variance followed by the Bonferroni *post hoc* test.

**Figure 7:**
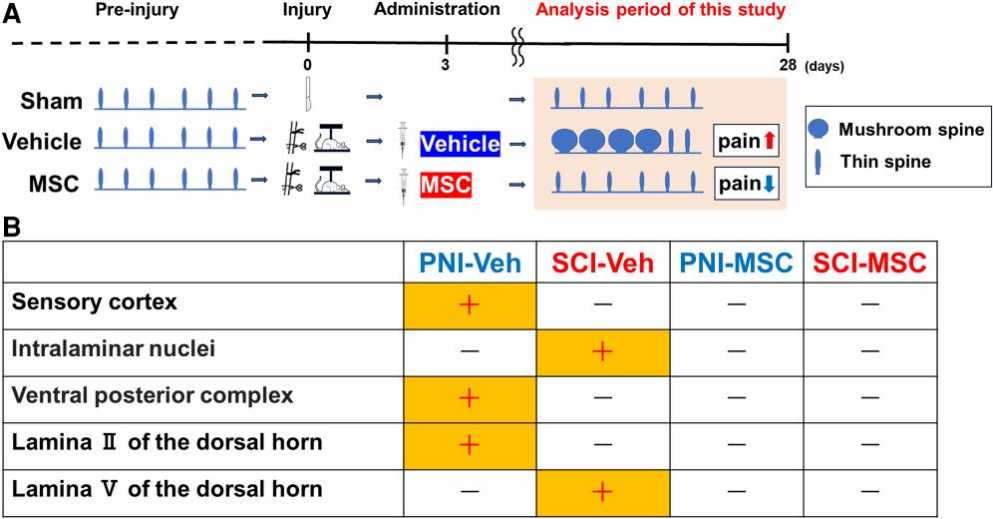
**Summary of the study.** Different injuries cause site-specific alterations in dendritic spines that are alleviated by infused MSCs. (**A**) Schematic illustration of the remodelling of dendritic spines (**B**) Summary of the morphological features of the mushroom-shaped dendritic spine. ‘+’ means that statistical significances were observed between vehicle versus MSC and vehicle versus sham. ‘−’means no statistical significance. MSC, mesenchymal stem cell; PNI, peripheral nerve injury; SCI, spinal cord injury.

We quantitatively analysed the density of total- (Figs. 4A-4–D-4 and 5A-4–F-4), thin-shaped (Figs. 4A-5–D-5 and 5A-5–F-5) and mushroom-shaped (Figs. 4A-6–D-6 and 5A-6–F-6) dendritic spines in neurones and the total (Figs. 4A-7–D-7 and 5A-7–F-7), thin-shaped (Figs. 4A-8–D-8 and 5A-8–F-8) and mushroom-shaped (Figs. 4A-9–D-9 and 5A-9–F-9) changes in the spatial distribution of dendritic spines of the three experimental groups (sham, vehicle, MSC) in both PNI (Figs. 4A and C and 5A, C and E) and SCI (Figs. 4B and D and 5B, D and F) models in the spinal cord (Fig. 4) and brain (Fig. 5).

### Differential remodelling of dendritic spines in the spinal cord following PNI and SCI

In the spinal cord of the PNI model, the density of mushroom-shaped spines in the PNI-vehicle group was higher than that in the PNI-sham group in Lamina II (Fig. 4A-6), not Lamina V (Fig. 4C-6), of the dorsal horn. Sholl analysis demonstrated a significant increase in the density of mushroom-shaped spines at any distance from the cell body in the PNI-vehicle group compared to the PNI-sham group (Fig. 4A-9). In the spinal cord of the SCI model, the density of mushroom-shaped spines increased in Lamina V (Fig. 4D-6) of the dorsal horn in the SCI-vehicle group, but not in Lamina II (Fig. 4B-6). Sholl analysis also revealed a significant increase in the density of mushroom-shaped spines near the cell body in the SCI-vehicle group compared to the SCI-sham group in Lamina V (Fig. 4D-9) of the dorsal horn.

### Differential remodelling of dendritic spines in the brain following PNI and SCI

In the brain of the PNI model, the density of mushroom-shaped dendritic spines in the PNI-vehicle group is higher than that of the PNI-sham animals in the SI (Fig. 5A-6). Based on the Sholl analysis, there was a significant increase in the density of mushroom-shaped spines at any distance from the cell body in the PNI-vehicle group compared to the PNI-sham group in the SI (Fig. 5A-9) and a significant increase in the density of mushroom-shaped spines near the cell body in the PNI-vehicle group compared to the PNI-sham group in the ventral posterior complex of the thalamus (Fig. 5E-9). In the brain of the SCI model, the density of mushroom-shaped spines in the SCI-vehicle group was higher than in the SCI-sham group in the intralaminar nuclei of the thalamus (Fig. 5D-6). Sholl analysis demonstrated a significant increase in the SCI-vehicle group of the density of mushroom-shaped spines at any distance from the cell body in the SCI-vehicle group compared to the SCI-sham group in the thalamus (Fig. 5D-9).

### Restoration of dendritic spine morphology following infused MSCs

Intravenous infusion of MSCs reversed pathological changes in dendritic spine morphology in the spinal cord and brain, so that dendritic spine dysgenesis was much less prominent in treated animals, in both the PNI and SCI models. This protective effect on dendritic spines was observed in Lamina II of the dorsal horn (Fig. 4A-6 and A-9), SI (Fig. 5A-6 and A-9) and the ventral posterior complex of thalamus (Fig. 5E-9) of the PNI model. In SCI, this protective effect was also seen in Lamina V of the dorsal horn (Fig. 4D-6 and D-9) and the intralaminar nuclei of the thalamus (Fig. 5D-6 and D-9) after systemic MSC infusion.

### Differential gene expression in the brain following infused MSCs

To investigate the possible mechanism of the neuropathic pain induced by PNI and SCI, we examined the mRNA expression levels of several α-amino-3-hydroxy-5-methyl-4-isoxazolepropionic acid (AMPA)-associated genes (*AKAP5*, Fig. 6A; *ACTR2*, Fig. 6B; and *SORBS2*, Fig. 6C) in the brain, which are related to dendritic spine dysgenesis, including an increase in mushroom-shaped dendritic spines.^46^ In the PNI model, the expression levels of these genes in the PNI-vehicle group were elevated compared with those in the PNI-sham group in the SI (*AKAP5*, Fig. 6A-1; *ACTR2*, Fig. 6B-1; *SORBS2*, Fig. 6C-1), not in the thalamus (*AKAP5*, Fig. 6A-3; *ACTR2*, Fig. 6B-3; *SORBS2*, Fig. 6C-3). However, in the SCI model, the expression levels of these genes in the SCI-vehicle group were elevated compared with those in the SCI-sham group in the thalamus (*AKAP5*, Fig. 6A-4; *ACTR2*, Fig. 6B-4; *SORBS2*, Fig. 6C-4), not in the SI (*AKAP5*, Fig. 6A-2; *ACTR2*, Fig. 6B-2; *SORBS2*, Fig. 6C-2).

Notably, the expression levels in the MSC group were suppressed compared with those in the vehicle groups in both the SI of the PNI model (*AKAP5*, Fig. 6A-1; *ACTR2*, Fig. 6B-1; *SORBS2*, Fig. 6C-1) and the thalamus of the SCI model (*AKAP5*, Fig. 6A-4; *ACTR2*, Fig. 6B-4; *SORBS2*, Fig. 6C-4). Taken together, these results suggest that intravenous infusion of MSCs suppresses AMPA signalling pathways in both the SI and thalamus in both PNI and SCI. All *P*-values and effect sizes in Fig. 6 are provided in Supplementary Table 2.

## Discussion

We investigated the effects of intravenous MSC infusion on neuropathic pain in both PNI and SCI models. We measured pain thresholds using the von Frey filament test for mechanical pain and the radiant heat test for thermal pain. In both models, neuropathic pain persisted after injury induction. MSC-treated animals showed a gradual and significant relief of neuropathic pain compared to vehicle-treated animals. These results demonstrate the alleviation of neuropathic pain induced by both injuries after intravenous infusion of MSCs. We did not observe increased thresholds indicative of sensory loss in our model system. To our knowledge, there is no direct animal literature parallel to human sensory loss.

A correlation between the altered dendritic spine morphology in the spinal cord and injury-induced pain has been reported in both PNI^3,4^ and SCI.^5,6,39^ In the current study, in agreement with previous work, we observed differential expression of mushroom-shaped dendritic spine remodelling in the spinal cord depending on the site of injury; specifically, Lamina II in the dorsal horn was affected in the PNI model, whereas Lamina V in the dorsal horn was affected in the SCI model.^4-6,39^ Importantly, intravenous infusion of MSCs decreased the mushroom-shaped dendritic spines in the spinal cord of both models, i.e. Lamina II in PNI and Lamina V in SCI, respectively. Considering these morphological alterations, these findings show that infused MSCs contribute to the reduction of neuropathic pain, regardless of the location of dendritic spine dysgenesis within the spinal cord, and may provide an effective tool for modifying pain due to both PNI and SCI.

In the current study, we have shown that not only the spinal cord but also the brain responds to injury by increasing mushroom-shaped dendritic spines on neurones, which are associated with neuropathic pain induced by PNI and SCI. Interestingly, altered dendritic spine morphology is site specific and occurs in specific regions of the brain in a pattern that depends on the type of injury. In the PNI model, the remodelling of dendritic spines into a mushroom-shaped spine in the brain was observed predominantly in the SI and the thalamic ventral posterior complex but not in the intralaminar nuclei of the thalamus. In contrast, in the SCI model, we observed dendritic spine remodelling mainly in the intralaminar nuclei of the thalamus, not in the SI and the ventral posterior complex of the thalamus. These distinct patterns of dendritic spine dysgenesis are consistent with previous electrophysical studies in the SI^9,10^ and thalamus^8^ after PNI and in the thalamus^47^ after SCI and provide a morphological correlate for this differential pattern of change within the CNS. Taken together, these observations suggest that even in the context of injury-specific changes in different brain areas, an increase in mushroom-shaped dendritic spines plays a fundamental role in the development of neuropathic pain. Infused MSCs reduced the neuropathic pain following both PNI and SCI and were associated with reduced spine dysgenesis, providing support for the idea that dendritic spines play an important role in the pathophysiology of pain following both PNI and SCI. Although our current regions of interest were primarily selected based on rodent physiological findings, future animal studies should explicitly integrate insights from recent human studies on additional CNS regions such as the secondary somatosensory cortex (SII), the posterior and anterior insulae, the anterior mid-cingulate cortex (aMCC), the supplementary motor area (SMA)^48^ and the pulvinar nucleus.^49^ Our observations also contribute to understanding the molecular signature of these structural profiles with mushroom-shaped dendritic spine remodelling . We performed qRT-PCR and found that the expression of cytoskeletal genes related to actin in the dendritic spine associated with the AMPA receptor (*AKAP5*,^46^  *ACTR2*^50^ and *SORBS2*^51-53^) was upregulated in the SI of the PNI model and the thalamus of the SCI model following injuries. This differential expression pattern was consistent with the structural signature of mushroom-shaped dendritic spine remodelling. Previous reports suggested that when a mushroom-shaped dendritic spine increases following injuries, the surface area of the tips of dendritic spines increases, which in turn increases the number of AMPA receptors, resulting in increased signalling and enhanced neuropathic pain.^4,6,43^ Interestingly, the current study shows that intravenous infusion of MSCs suppressed the increased AMPA-associated gene expression following injuries, suggesting a molecular mechanism that might result in reduced neuropathic pain in both PNI and SCI models.

A limitation of this study includes the exclusive assessment of male rats. Male animals were selected to maintain consistency with our previous studies^3,6,39^ and to minimize biological variability associated with hormonal fluctuations, which may affect both pain response^54^ and the therapeutic efficacy of MSCs. However, given the growing evidence of sex differences in pain mechanisms,^54^ the lack of female subjects represents a critical limitation. Future studies should include both male and female animals to provide a more comprehensive and translationally relevant understanding of sex differences in pain responses and the therapeutic efficacy of infused MSCs in PNI and SCI models. In addition, we employed only reflex-based behavioural assessments. This may limit our ability to evaluate the affective/emotional dimensions of pain, which are critical for translational relevance. In future studies, operant paradigms (e.g. learned escape, place aversion and reinforcement conflict) and assessments of pain-affected complex behaviours (e.g. anxiety, attention, disability, sociability and sleep) using appropriate and validated methods should be considered.^55^

Despite this limitation, our observations in the rat of the differential effects in the brain and dorsal horn between the PNI and SCI models suggest that these injury types may involve fundamentally distinct mechanisms; it remains to be determined whether these rodent models recapitulate the clinical reality of human neuropathic pain.^15,16^ There might exist differential pathways in the brain associated with neuropathic pain.^56^ The PNI model extends the connection to the SI from the ventral posterior complex of the thalamus. In the SCI model, the path might connect from the mediodorsal nucleus within the intralaminar nuclei of the thalamus from the spinal cord. Irrespective of underlying mechanistic differences, our findings of injury-specific alterations in dendritic spine morphology in the dorsal horn and brain regions may reflect how distinct pathways and cellular responses contribute to pain processing in each model. Importantly, we have also demonstrated that systemic administration of MSCs alleviated both PNI- and SCI-induced neuropathic pain, which may be related to the preservation of relatively normal dendritic spine morphology within multiple CNS regions affected regardless of PNI or SCI. The intravenous route of MSCs may be advantageous in this respect because the cells or their released contents have access to both the spinal cord and brain (Fig. 7A). Further studies into the mechanisms related to neuropathic pain following PNI and SCI may help to inform the development of therapeutic strategies for different types of nervous system injuries.

In conclusion, we have demonstrated that intravenous infusion of MSCs significantly alleviates pain behaviours in rat models of neuropathic pain induced by PNI and SCI. We observed distinct patterns of dendritic spine remodelling in two brain regions (thalamus and SI) involved in pain processing in these two rodent pain models (Fig. 7B), reinforcing the structure–function relationship between dendritic spine dysgenesis and abnormal pain and introducing the possibility that, in some species, different forms of pain result in different topographic patterns of dendritic spine remodelling within the brain. Additionally, these morphological profiles in each pain-injury model correlated with alterations in AMPA-related gene expression. Overall, our findings suggest that systemic MSC administration holds promising therapeutic potential for alleviating neuropathic pain across various injury types and affected CNS regions via a mechanism that includes preservation of relatively normal dendritic spine morphology both within the spinal cord and brain. Future studies should elucidate additional signalling pathways, cytoskeletal molecules and synaptic proteins,^57,58^ particularly those associated with different molecular expressions underlying distinct dendritic spine morphologies. In our PNI model, MSCs were administered after confirming the onset of allodynia on Day 3 using von Frey and radiant heat tests. Another study demonstrated that mechanical and cold allodynia in their SNI model emerged within 24 h and persisted for at least 6 months.^59^ The relationship between the development of allodynia and the temporal course of dendritic spine dysgenesis has not been fully elucidated and warrants further investigation. Intravenous infusion of MSCs facilitates functional recovery in both acute^25^ and chronic^24^ phases of SCI. Thus, infused MSCs might exert either preventative or curative effects on neuropathic pain; these aspects should be clarified in future studies. As intravenous infusion of auto-serum-expanded autologous MSCs derived from the bone marrow has been shown to be safe and potentially therapeutic in patients with acute^60^ and chronic^61^ SCI, it might be of interest to determine whether neuropathic pain in human patients is also ameliorated following MSC infusion in the future.

## Supplementary Material

fcaf494_Supplementary_Data

## Data Availability

The data that support the findings of this study are available from the corresponding author upon reasonable request.
